# A Report of 25 Cases of Pneumonia Treated with Pane’s Antipneumonic Serum1Read before the Section of Medicine of the Buffalo Academy of Medicine March 6, 1906.

**Published:** 1906-06

**Authors:** Giuseppe Tartaro

**Affiliations:** Buffalo, N. Y.; 129 Court Street


### BUFFALO MEDICAL JOURNAL.

VOL. LXI. JUNE, 1906. No. 11

### ORIGINAL COMMUNICATIONS.

### A Report of 25 Cases of Pneumonia Treated with Pane's Antipneumonic Serum.[1]

BY GIUSEPPE TARTARO, M. D., Buffalo, N. Y.

THE ideal therapy of infectious diseases is serumtheraphy. Science will have the last word when, after the finding of the specific pathogenic agent of a contagious disease, will find a substance to fight it, and its toxins in the system. Serum therapy has already triumphed over diphtheria and anthrax, and is getting ready to overpower bubonic plague, erisypelas, tetanus, pneumonia, syphilis, cancer, etc.

Speaking only of pneumonia, I would say that notwithstanding the restless search for new drugs, methods and presidia, the old theraphy did not progress much. The percentage of deaths is just as high now as it was fifty years ago, and without being sceptical, we can say that our therapy of pneumonia which is aiming to fight the symptoms, it and not the cause of disease will not last long. Still we must use it, because there is nothing better. We will now see how far we are from this problematic stage which is our longing, and what is better today.

While legions of modest and patient investigators are at work, the result of their studies is worth knowing, be it crowned by success or not, because the practitioners are the judges. You hear of a serum therapy of pneumonia, and often you have heard criticisms against it. I claim that those criticisms are unjust on account of the use of serums prepared for commercial purposes, which are lacking of nearly every curative property. So, it is only right that you hear tonight a report of cases treated with the original and true antipneumonic serum which was discovered by a very modest worker to whom a good deal of damage has been done by his own modesty, and by the absence of any pecuniary reward, and perhaps by his own place of birth.

---

1. Read before the Section of Medicine of the Buffalo Academy of Medicine March 6, 1906.

After the two Klemperers found antipneumonic power in the blood serum of convalescents from pneumonia, Emmerich succeeded in immunizing animals against pneumonic infection, and afterward the Klemperers, Foa, and others did the same. But it was only in 1896 that Nicola Pane, of Naples, stated the finding of a serum which, inoculated in progressive doses to rabbits, gave them power to stand injections of doses of pure culture of diplococci 60 millions times higher that the minimum dose capable of killing a not immunized rabbit. Then the serum was tried on men in De Renzi's clinic in Naples and found satisfactory. The same trial was given in other Italian clinics with differing results. Sometimes the malice or jealousy of men or schools operated the failure of the undertaking which should have had a frank and impartial trial from scientific men. However the sum of Italian clinics' judgment is, that Pane's serum has real power against pneumonia.

Maragliano of Genoa says that it leaves unchanged the local symptoms, but it improves the general symptomatology and causes the fading away of toxinic notes even if very marked, lowers temperature and hastens crisis. Special researches showed increased leucocytosis. Massolongo found Pane's serum useful in pneumonia of patients arthritic, nephritic, cardiopatic, alcoholist, and of old age, and acting very favorably by opposing the diffusion of the process and making easy the resolution. De Renzi found general improvement, lowering of temperature, pulse's and breath's rate, and sometimes the end by lysis.

Pane makes the serum of two strengths. The first is simply immunized donkeys' serum plus 25% of kresol. The second is two or three times stronger than the first, and is to be used in more serious cases or in well advanced stages of the disease. Pane suggests the use of the serum as soon as posssible either 20 ccm of No. 1, or 10 ccm of No. 2 and to repeat the injections every 12 hours until the sickness ends.

In ordinary cases 10 or 20 ccm *pro die* are enough. But this dose may be increased in very severe cases to 100-120 ccm *pro die*, the serum being completely harmless. Serum therapy of pneumonia does not require the help of any other medicine except 30 ccm of alcohol *pro die* diluted in water, or some wine. The serum is injected in the subcutaneous tissue of the back along the posterior axillary line. In very severe cases it has been injected into veins at a dose of 5-10 ccm. Marigliano increases the dose of serum *pro die* according to the time of the beginning of treatment, and severity of symptoms, and keeps on injecting 10 ccm every 12 hours until temperature falls below 38.5° cg., stopping then, but again injecting whenever there is a new rise. In most severe cases 100-120 ccm *pro die* has been used by Pane himself in the 5th or 6th day of the illness. But serum is absolutely useless, whatever be the dose, in the preagonic period. Serum does not lose any power within a year or so. I have had very good results from serum eleven months old.

Within 3 years I gathered together 25 cases of serum therapy of pneumonia of which I include 4 cases of lobular pneumonia to show that in this disease too, Pane's serum may be useful, as it may be in diplococcic meningitis, ulcus serpens of the eye, and in every other localisation of pneumonic infection. Of those mentioned, 3 are cases treated by Dr. Hartwig; and one a case that Dr. A. W. Hengerer treated for me. I used always Pane's serum No. 2, rejecting every other serum. In Italy now, Tizzoni and others are experimenting with different serums, but their results are very unsatisfactory if not a complete failure. In Germany, Merck is trying to make some serum under the direction of Romer of Wurzburg, to be used against ulcus serpens, that is a diplococcic infection, but the results are still unknown and beside the only object of the treatment is the ulcus serpens.

In the United States, Mulford now makes antipneumonic serum with Pane's name, saying that it is made after his methods, but Mulford's serum has been tested on animals by Pane himself in his own laboratory, and has been found useless. It gives abscesses sometimes. Pane never gave any permission for making his serum.

Although there are statistics of nearly 800 cases of serum therapy of pneumonia both by American and foreign physicians with various results due mostly to the different kinds of the serum used, I would prefer to say something about my own experience, being unable to trace in the satistics the qualities of the make of serums. Italian physicians of New York city using Pane's [illegible]hentic serum, and other physicians of this country are claim-[illegible] good results, so that I would be very glad if anyone of my colleagues would give Pane's serum a trial.

In my 25 cases I did not follow Pane's rules of injecting 10 ccm twice *pro die* until the disease terminated, or at least until a temperature of 100° is reached. As a rule I made a single injection in the course of the sickness. In one case I made two, and in one of Dr. Hartwig's cases he made two. The price of serum is rather high and the occurrence of the disease in people of very limited means forbids me being very generous in the use of serum suggested by Pane, and however insufficient the dose of 10 ccm might seem according to Pane's ideas, I find that satisfactory in the majority of cases.

With the serum, I never used any medicine whatever, no strychnia, no nitroglycerine, no baths, no antipyretics, no cold applications, but from time to time a very weak expectorant. I used freely wine, or alcohol for people accustomed to it, and the usual diet of pneumonics, light but nourishing. In this report I limit myself to clinical observations. The microscopical observations I made, are very few, and were made in haste, and not in every case, so they cannot strengthen my conclusions. Still I hope to be able in the future of demonstrating only with the microscopical observations of sputum before and after serum therapy to explain why the serum succeeds or does not, and what is the reason of different results. In 25 cases I have had only two deaths occur. Do not understand from this that I make any reference to a death rate, 25 cases being insufficient to make rules of percentage.

One case was a child 16 months old with lobular pneumonia, secondary to measles. The injection was made only 5 hours before the death, when there was already a very marked bronchial paralysis, and therefore I did not expect any good result from the serum. A brother of this child, 4 years old, had previously lobular pneumonia too, was benefitted from one injection of 10 ccm of serum to the extent of a lowering of 2½ degrees of temperature in 24 hours. This lasted nearly 40 hours, then the temperature went up again for 18 hours, and finally the illness ended by crisis.

The second death occurred in Irving, N. Y., under conditions worth explaining. It was a lobar pneumonia in the fifth day. I was called in consultation, and found the patient doing well, with good pulse, nearly no cyanosis, rather dispneic, and with temperature of 103 2-5.° I injected the serum and returned to Buffalo. That was at eleven o'clock in the morning. In the afternoon the patient showed a fair improvement, felt stronger and comfortable, was brighter, less dispnea, talked pleasantly with his relatives. His physician saw him again at 4 o'clock and noted the improvement, but at 5 o'clock occurred a fire at the patient's house, and in the panic he was roughly dragged out of his bed, not properly clothed, was hurriedly wrapped in sheets and taken to a neighboring house. It was a cold day in March and his condition changed to the worse after one hour; six hours later he died. I do not make any comments.

For the sake of brevity I will show you three clinic charts which give the three typical courses of pneumonia under serum therapy. The first is the most common occurrence.

A strong laborer while working has a chill, feels unwell, goes home and to bed. He has fever, pain in one side of the chest, cough, and the sputum is but slightly colored. The second day and even the third day, when the sputum is markedly red and cough and fever are still going on, the physician is called. He finds the complete symptomatology of lobar pneumonia. Ten ccm of serum is injected. After 8 to 12 hours the symptoms are changed, the patient that was restless, dispneic, uncomfortable during it all, now feels comfortable. That is the most important thing for himself. He feels born to a new life, the temperature drops 2 to 2½ degrees, the pulse rate is slower, the pulse is regular, the dispnea has diminished; the sputum fluider and less rusty. Still the dulness of the affected lobe is unchanged, but the most important thing is that the patient feels much better. Some patients at this time deem it right to dispense with the physician's attendance.

The fever goes on markedly low for several other days, while the patient's condition remains very good, there comes a crisis almost unworthy of that name, the disease is past, and convalescence begins. This happens in nearly three quarters of the cases.

The other type of clinical course second in order of frequency is that in which a man is taken sick in the same conditions of the first, repeating nearly the same nosological table, but with marked cardiac irregularity and where there has been a much more belated intervention of serum therapy, to wit: in the 4th or 5th day. These are the cases where a larger dose, than 10 ccm of serum only once, to be effective is necessary according to Maragliano, and where injection *bis in die.* must be repeated until temperature is kept steadily below 38.5° centigrade. In these cases just as in diphtheria with only 10 ccm of serum, very seldom one can lower the temperature, or if so, it is only for a few hours, say 8 to 12, and then temperature rises again. But, notwithstanding the very small dose, one finds that the patient is relieved, and does not worry that the fever is still high; he feels better, cyanosis is gone, sometimes pain too is gone; the local objective symptoms, dulness, rales etc., are unchanged. In due time the crisis comes and the sickness is all over. Of the changes caused by the serum, there is no record in the clinic chart, only the bulletin registers "patient feels better."

It may seem very little or nothing to us, but the patient will never forget that feeling of relief that is the only manifestation of a very important fact, which we are unable to register in our clinical charts, made of numbers and lines, but which is nevertheless the expression for the putting out of combat of all pneumonic toxins accumulated in the system during 4 or 5 days of illness, and of the increased leukocytosis, and of the fight of anticorps against pneumococci.

The third type is rather rare in full grown men. I have seen it only twice in them, but it occurs in children very often. A pneumonitic in the third day with very high fever, for instance 105 degrees, receives the injection of 10 ccm of serum. After 12 to 18 hours the temperature falls to 100 degrees, breathing becomes nearly normal, pulse rate is diminished; twelve hours later bowels move very freely once or twice, temperature drops to normal point and disease ends by crisis at the fourth day.

These are fundamental types of pneumonia's course under serum treatment, which come in order of frequency. But there are other cases differing from this usual routine, and not less interesting. A woman 32 years old is taken sick, and the same day calls the physician (this is Dr. Hartwig's third case) who finds lobar pneumonia with very marked cardiac troubles, threatening seriously the patient's life, already, since the beginning of illness. Still temperature was low (rectal 101 2-5°, pulse 118, weak and irregular, respiration 33). Ten ccm of serum was injected, (this is the only case I have of injection in the first 24 hours). Within two hours the temperature dropped 3-5 and in 12 hours reached a minimum of 98° (a drop of 3 2-5°), pulse 96, respiration 24.. The pulse becomes stronger and more regular, and the patient feels so much relieved, as to impress wonderfully both the attending physician and the family. Twelve hours later the temperature rises again to 101°, but the dispnea is not so marked as before, from 33 dropped to 20-24-22 and remaining so for the duration of illness. The pulse too, jumps from 105 to 120. A second injection of 10 ccm in the third day causes a drop of only 1 degree of temperature, but the patient feels comfortable. The sickness ended the seventh day by crisis. This case is noteworthy on account of the injection made in the very first day of illness, causing a considerable drop of temperature, checking the most alarming symptoms and changing to a rather mild case, one which since its very beginning threatened. with cardiac complications, a fatal end.

Besides, the second injection did not lower so much the temperature, as the first one did, but helped to make more uniform and regular the functions of heart and lungs. Dr. Hartwig coincides in emphasising the improvement following the first injection.

Another case—a very strong man, 31 years old, with pneumonia in the second day. His peculiarity was a sputum bright red, frothy, hemorrhagic, and very plentiful, (within 4-5 hours over 4 fluid ounces), temperature 103 3-5°. Ten ccm of serum are injected in the morning of the third day. Within 12 hours temperature drops to 101 1-5°, and what is more noteworthy the sputum loses its hemorrhagic character, becomes dark like plum juice, and decreases to less than one ounce in 24 hours. As usual the patient is relieved, hence I do not inject any more serum, but the fever keeps on steadily between 101½° and 102 3-5° and in the seventh day comes the crisis.

It is useless to speak about the other cases, which are more or less like the ones referred to. Commenting now on all the cases, I wish to keep myself within the ground of clinical facts for reasons already given, and for the sake of clearness I will make comparisons from time to time with diphtheria serum therapy and its characters familiar to all.

The facts perfectly ascertained from clinical side, in my 25 cases are:—

I. Percussion and auscultation of a lobe affected with pneumonia, before and after the injection of serum, does not show any change. Dulness remains and in a very few cases the exudate seems more fluid.

II. Serum mitigates the gravity of pneumonia, but as a rule does not shorten its course except in children. The two cases I had of crisis in fourth and fifth day, strictly speaking, are possible in pneumonia without serumtherapy, and in these peculiar cases we cannot either assert or deny serum's action.

III. A drop of temperature of one to three degrees and even four degrees is caused by the injection of 10 ccm of serum. The earlier the injection, the more marked, as a rule, temperature's decrease. This is like diphtheria. It seems that in belated cases a much larger and stronger dose of serum repeated at 12 hours interval should be successful; this too is already practised in diphtheria, and there is no reason why it should not be done in pneumonia, unless it be for economical reasons.

IV. Serum causes the subjective phenomenon of relief nearly in every case, even when there is not any change of temperature. This seems to be due to antitoxinic properties of serum, but moreover as Pane asserts, to production of anticorps and to increased leukocytosis. The role of anticorps in fighting the pneumococci or diplococci in the cells of the system has been already demonstrated by Pane.

V. Cyanosis even very marked disappears after the use of serum. Albumin when not due to pre-established conditions of kidneys disappears after the use of serum.

VI. Cardiac complications in pneumonics due to pneumonic toxins are relieved by the serum as every other complication due to pneumonic toxins.

As stated before it would be rather difficult to find out why Pane's serum gives these results, and I may venture to make some conclusions. I have no pathological anatomy observations of cases treated with serum, and perhaps there are few, or none at all in the rather lengthy bibliography of the subject, but I have no doubt that there are considerable marked changes that ought to take place in the lung tissues after the injection. We cannot find out with the known means of physical examination any change; the dulness is the same after the injection as it was before. But it will be easy to explain our failure.

In diphtheria we see the softening of membranes due to antitoxin, but we see too that their location remains hyperemic for sometime, and we understand that anatomical alterations of mucosas and bloodvessels cannot disappear very quickly. But we ought to allow a reasonable length of time for the restitutio ad integrum. Still, diphtheric localisations are very small if compared with pneumonic localisations, and their pathogenic agent far differing from microbiological point of view, and far simpler too. In this way we may understand why dulness and rales cannot disappear so quickly had the serum still been used, and why restitutio ad integrum of an affected pulmonary lobe must require much more length of time than in diphtheria. For instance: considering the size of the affected organ, the difficulties it meets in freeing itself from its pathological products, and the anatomical alterations it went through.

I tried to keep in touch for some time with my patients after their dismissal and in a few cases where I succeeded in seeing them, often for a period of two or three months after illness, I found them showing still traces of the late disease, sometimes with rales or coughs and sometimes a special liability to feel pain in the chest at every change in the weather. It impressed me that every patient that showed at the time of illness very marked good effects of the serum, got rid quicker of the secondary troubles. The other ones where serum therapy was used in more belated period causing very little or no changes at all in temperature, showed for a longer time traces of the late disease.

A complete, exhaustive, microscopical examination of sputum in every case, and in every day of illness will show a good many remarkable points in the pathological anatomy of pneumonia when under serum treatment, and will show besides where lays the cause of some so called failures of serum: because it will show sometimes bacteric associations upon which we have no power at all with our serum made only for the purpose of fighting pneumonia when given by Frankel's diplocci or Friedlander's pneumococci, or cases of pneumonia due exclusively to other bacteria that the said before, while we will see a pneumonia due purely to pneumonic infection, give up quickly when confronted with Pane's serum.

Pane's theory is that pneumococci and diplococci are of the same family, and he is making his serum as an antipolipneumonic serum, not as an antipneumococcic, or as an antidiplococcic. This is the reason why he calls it antipneumonic serum.

Considering now the patient's feeling of relief or euforia after the injection, we can guess it is possibly due partially to its antitoxinic action, and from that we might perhaps find out if the bacteric toxins freely circulating in the system may account at all for keeping up the fever. But, as I said before, all this is mere guessing. The principal objections to Pane's serum are:

1st. It does not cut short the disease as diphtheric antitoxin so often does. I answer that there are far different diseases and we cannot force the results of pneumonia's serum therapy into the rules already fixed for diphtheria. Pane remarks that diphtheria is a toxinic infection and pneumonia a bacteric infection.

2nd. That Pane's serum does not seem to have any power upon the disease's local manifestations. As I said before, we have not enough knowledge of pathological anatomy of cases treated with serum.

3rd. That the result of serum therapy is uncertain, and not the same in every case. This objection loses its ground when you think of the different pathogenic agents or associations of agents capable of producing lobar pneumonia, and the unicism of cultures employed in the production of serum; and when we think of the different use of serum in the different stages of the disease.

In conclusion, I would say that the therapy of pneumonia ought to be brought to a more rational method by the adoption of serum therapy, which adapts itself to all indications of the various, complicated, and often deceptive therapy we are using now, by its being antithermic, cardiocinetic, antidispneic and antitoxinic. Goldsborough says, "I should feel culpable to a great degree, had I a case of pneumonia and failed to use the serum." Because he would fail to help the patient with a remedy that is the best and the surest.

So we cannot dispense with serum therapy of pneumonia however incomplete one might deem its activity when considering its action summarily without taking the pains of investigating the bacteric nature of every case of pneumonia one has to fight.

In the practice there may be some failures, but we look hopefully toward the future which may improve this remedy so as to make it generally adopted. Certainly it does not do to be sceptical to the lovers of this still child science of serum therapy, of which we see now only the most promising bud of childhood, while instead the next generation will certainly see the gorgeous blooming of the most beautiful womanhood.

129 COURT STREET.

